# Some Points in Rectal Surgery*Read before the Tri-State Medical Society, Atlanta, Ga.

**Published:** 1895-01

**Authors:** J. M. Mathews

**Affiliations:** Louisville, Ky.; Professor of Surgery and Diseases of the Rectum, Kentucky School of Medicine; 923 Fourth Avenue


### SOME POINTS IN RECTAL SURGERY.*

BY J. M. MATHEWS, M. D., LOUISVILLE, KY.,

Professor of Surgery and Diseases of the Rectum, Kentucky School of Medicine.

I feel deeply obligated to the members of this Society for its kind invitation through the worthy Secretary to read a paper at this session. The few remarks that I have to make, I shall confine to the subject of *Rectal Surgery*.

About eighteen years ago I had the honor of inaugurating a move to place rectal surgery upon the basis of the other specialties. I am more than gratified now, after the elapse of this time, to be able to say that it is distinctively recognized in this country and in Europe as a legitimate specialty. Time was when these diseases were principally treated by the charlatan, itinerant, and quack. To-day some of the leading surgeons in nearly every State in the Union are giving this study special attention. Whereas it has been but a short time ago that the attention given to rectal diseases was very meager, we see now original research in this department reported in almost every medical journal, and by some of the ablest surgeons in the profession. We can all remember that the time was when very little surgery was done for the hemorrhoidal disease, the practitioner being content with treating it medically. Since interest has been aroused in this line, surgeons have vied with each other in their attempt to demonstrate which operation is preferable for hemorrhoids. Even the clamp and cautery plan and the ligature have been superseded in the minds of some who have followed Mr Whitehead's advice, and who resort to the removal of the entire "pile-bearing" area. This operation, however, has not received much recognition in America, most all surgeons agreeing that it is too much surgery where a less amount will suffice. The carbolic-acid-injection plan has been relegated to the rear, the principle of which was opposed by all scientific surgeons. It was fraught with much danger to life and caused much local distress, was soon abandoned by the intelligent profession, and is now mainly used by the advertiser as a catchpenny. Of all diseases pertaining to the

*Read before the Tri-State Medical Society, Atlanta, Ga.

rectum fistula in ano is one of the most important. Before the days of this specialty the method practiced for its cure, even by the surgeon, was very crude and limited. Even our best text-books on surgery made it sufficient to say that all that was necessary to cure this affection was to pass a grooved director through the sinus and divide the structures thereon. In contrast to this Mr. William Allingham says, that to cure a complicated case of fistula in ano requires more delicate and precise surgery than most any other surgical affection of the body. All of us who have had to deal with severe, complicated cases of this disease can attest the truth of Mr. Allingham's remarks. In regard to the surgical treatment of the same, it must be admitted that the knife takes precedence over every other plan. The elastic ligature, it is true, in the past has received considerable attention, and from such high authorities as Littell, Allingham, and others, but the test of time demonstrates that but few cases of this trouble can be cured by this means. The injection plan of sinuses, as practiced by some, is not worthy of consideration. *Irritable ulcer, or fissure*, of the rectum has received a good deal of attention lately by such men as Harrison Cripps, Adler, and others. The latter is inclined to believe that many if not the majority of these cases can be cured by local treatment. If one will take the time to consider the pathology of this affection, it will, I think, be conceded that a simple divulsion of the sphincter muscle, which invariably cures all such cases, is much to be preferred to temporizing with local treatment. I desire to mention here that I never divide the muscle after the manner of Boyer, nor do I break the muscle after the manner of many surgeons. Neither, in my mind, is ever necessary, and by practicing such methods the office of the muscle is interfered with, if not entirely abolished. A great deal of attention has been given in the last ten years to the subject of the *reflexes* in a general way. And, too, specialists in every line have written much in relation to the reflexes in a special way.

At the International Medical Congress at Washington I had the honor of reading a paper, the title of which was, "The Anatomy of the Rectum in Relation to the Reflexes." In that paper I endeavored to show that much elucidation could be thrown upon many suspected diseases by tracing their origin to disease in the rectum. I meant only to convey the idea that many reflex symptoms could be made manifest through the nerve distribution from the affected part to distant parts of the body. The idea herein inculcated has been run away with in a wild manner by the so-called orificial surgeons, who are in the habit of removing an inch or two of the rectum for the most trivial cause of self-imagined reflex. I wish here to enter a most vigorous protest against this abominable practice. Such practice can not be too severely condemned by the medical profession, for in its wake lie many wrecked and wretched bodies.

One of the most important subjects to the rectal surgeon is *Ulceration of the Rectum.* It is a question often of very serious import, and one that staggers the physician, to determine whether it is malignant or non-malignant. I desire to say that simple ulceration of the rectum, from whatever cause, of any magnitude, is very seldom met with in my practice. In my book on "Diseases of the Rectum," I state it as my belief that sixty per cent. of stricture of the rectum, with or without the co-incident ulceration, is caused by syphilis. In said work I use these words: "Indeed so firm am I in this belief, that if it is a question between cancer or no cancer, and it is decided that it is not malignant, ninety-nine out of every hundred cases will, in my opinion, prove to be syphilitic." This opinion, in its entirety, has been challenged, especially by one authority in America. In addition to saying that my percentage of syphilitic stricture is too large, he persists in saying that I believe that ninety-nine out of every hundred cases of stricture of the rectum are syphilitic. I would beg anyone who is interested in this subject, to turn to page 346, chapter 15, in my book, and read the following: "Stricture, the result of benign ulceration, does not resemble in the least stricture from malignant causes. To the contrary, syphilitic stricture does to a degree resemble malignant growth. To be plainer, malignant disease and syphilitic disease invade the rectum as a deposit and infiltration of the sub-mucous tissues, etc. Ulceration here is secondary to the deposit and is caused by the friction of the passage of fæces, or the breaking down of tissue, the result *of* the disease. Benign, or simple ulceration, begins with the damage done to the mucous membrane, and the infiltration is secondary to it, unlike both malignant and specific disease. Besides, a simple stricture is generally annular, and does not consist of a deposit in the submucous tissue. I do not wish to convey the idea that ninety-nine out of every hundred cases of stricture of the rectum are syphilitic by any means, but I have been thus explicit because I have been quoted wrong in this matter a number of times." It does appear that these words should make my meaning clear, but it does not seem to have done so with the authority mentioned.

*Cancer of the Rectum* is, of course, all important to the surgeon. I have always maintained that where it was practicable, it is better to extirpate the growth than to resort to colotomy. By the admirable device of Kraske we are enabled with much less trouble to remove the rectum and much more of it than by any other method, and he is to be congratulated upon his admirable operation. The time that I should occupy before your society will not permit me to deal extensively with this subject, and I only desire to say that in my opinion the surgeon who attempts the operation of removing the rectum under any other condition than that the growth can be circumscribed, and no infection exists, is guilty of wrong practice. I also desire to say that I believe, if the majority of cases in which colotomy has been done could be investigated, that upon a thorough reasoning and scientific basis, they could be pronounced unwarrantable. Much odium has been brought upon the medical profession for doing unjustifiable surgery, and in no department is this accusation founded in more truth than where many operations have been done upon the rectum and the colon. I wish I had the time to mention a number of operations that are new to rectal surgery, which would tend to prove to you the wonderful advance that has been made in this department in the last few years.

In this connection I desire to report three cases that came under my observation last week. I desire to call attention to the fact that three different methods were practiced in so many cases.

*Case* 1. Mrs. E., age thirty-five, was sent me from a southern city, and gave the following history: About two years prior to her coming, she complained of pain in the rectum, radiating to the back, thighs, and ovarian region. This continued to increase for one year, when she went to a sanitarium for treatment. The physician in charge did an operation upon the cervix, and also attempted to repair a recto-vaginal fistula. She was kept in the institution several months without any appreciable benefit. She returned home, and after the lapse of six or eight months she came to me. Upon examination I found an ulceration that had completely infiltrated the rectum involving the sphincter muscles and extending into the vagina. Its character was clearly malignant. The pain incident to the disease was unbearable. I at once suggested a colotomy to her, which operation was performed a few days after her arrival at the infirmary. Very great ease was afforded her by the operation. She sat up on the fifth day, and went home at the end of two weeks.

*Case* 2. Mrs. B., a young married woman, age twenty-three, was referred to me by a local physician. She gave the following history: About three years back she complained of pain in the rectum, with a thorough constipated habit; the pain, however, was never a factor in her case, but the constipation grew worse, and could in the last six months be called an obstipation, for the reason that she could scarcely have an operation from the bowels at all. During this interim she had consulted several physicians, two of whom had attempted at different times to close a recto-vaginal fistula. Both attempts had failed, and the surgery upon the perineum had left that portion of the anatomy in a shattered condition. Upon an examination with the finger, I found extensive ulceration of the rectum, and a close stricture about one inch above the external sphincter muscle. The character of this ulceration was plainly syphilitic. I told the woman that this was my opinion, and she confided to me the fact that she was a courtesan before her marriage, and had had syphilis. I should add, that notwithstanding this extensive ulceration and close stricture, which would not admit the point of the little finger, this woman was in splendid physical condition and apparent good health. The process of gradual dilatation was made twice per week at my office by means of a speculum until, after eight or ten trials, the dilator was opened to its full capacity.

*Case* 3. Mrs. H., age forty, a widow, was referred to me, with the following history: For about four years she had suffered with obstinate constipation, and for the past eighteen months had passed blood and mucus and pus *per rectum*. She was feeble and emaciated and very melancholy. Believing that the ulceration was beyond doubt syphilitic, the fact was ascertained by questioning, that her husband had died six months before in an insane asylum from a syphilitic brain trouble. Upon examination, I found in connection with the ulceration and stricture two fistulous tracts which had opened externally. I advised her to go to the infirmary, which she did, where I resected the stricture and laid open the fistulæ. The bleeding was so excessive in this case that the rectum had to be plugged after the operation.

It will be observed, that in the first case, I did the operation which I have condemned in this paper, namely, colotomy. My reason for so doing was that pain was a great factor in that case, and that the cancerous ulceration extended to the vagina, through which she passed most of her fæces. In the second case I practiced gradual divulsion, although in my work on "Diseases of the Rectum" I condemn this method. It will be observed, however, that I used a dilator instead of bougies, and took only eight sittings to freely break the stricture. My reasons for employing a gradual method instead of thorough dilatation at one sitting was that the perineum had been so injured by former operations as to render careful watching necessary for fear of further injury. In the third case I practiced dissection of the stricture. My reason for this was that it was clearly within reach and the rectum above not much invaded.

One of the most important things connected with such cases is the after-treatment. Where the rectum has been resected after the manner of Kraske and others, or where the lower portion of the rectum has been removed, it is necessary to guard the patient against the fæcal evacuation. Therefore it becomes of the utmost importance to look to the feeding and sustenance of the patient during perhaps a long convalescence.

Early in my career as a rectal specialist, I relied upon controlling this feature by putting the patient upon the milk diet.

I soon learned that this had objectionable features, so I was forced to look around for a substitute. I wish to say in this connection, these enfeebled patients who have undergone a severe operation upon the rectum, need often a stimulant as well as food. This was never more clearly illustrated to me than during my treatment of case No. 1. This woman was not only greatly enfeebled and emaciated, but she was given to the morphine habit. It occurred to me that she needed special medication, and I hit upon the idea of putting her upon the preparation of maltine with coca wine so strongly recommended by Dr. Henry C. Coe, of New York. By this combination I got not only a good food in the maltine, but an excellent stimulant with coca wine. Not only did it subserve these two purposes, but at the end of two weeks she was only taking one fourth a grain of morphine in twenty-four hours, instead of nine grains per day which she had been taking for months.

*923 Fourth Avenue.*

---

The Murphy Button.—Dr. Dawbarn said that about the time when the Murphy button was first used in New York, he sent to Chicago and got one which he used in a case of gall-stone, the diagnosis having been confirmed by the operation. The gall-bladder seemed remarkably friable, and at autopsy—death having occurred in forty-eight hours—a distinct tear was found at the edge of the button. There was also a diffuse form of cancer of the liver, and this had probably led to the friable condition of the gall-bladder, although this viscus was not carcinomatous. He had sent an account of the case to Dr. Murphy before the latter had reported a collection of one hundred successful cases, and had afterward written Dr. Murphy to learn why this fatal one had been omitted. The answer was that he did not suppose the author of the case would care to have it reported since it was unsuccessful, and also because it was an attempt to unite a cancerous gall-bladder with the duodenum, which was not true—From report New York Academy of Medicine in *Medical Record.*

